# Hidden in Plain Sight: Ralstonia mannitolilytica as a Rare Cause of Urinary Tract Infections

**DOI:** 10.7759/cureus.70013

**Published:** 2024-09-23

**Authors:** Sakshi Upendra Bhatia, Radha Kumar, Vidhyasagar Krishnamoorthy, Jai Durairaj Paramasivam

**Affiliations:** 1 Paediatrics, Saveetha Medical College and Hospital, Saveetha Institute of Medical and Technical Sciences, Saveetha University, Chennai, IND

**Keywords:** hydroureteronephrosis, posterior urethral valve, ralstonia mannitolilytica, urinary tract infection, vesicostomy

## Abstract

Children admitted to hospitals have been shown to frequently suffer from urinary tract infections (UTIs), with *Escherichia coli* being the most common cause. *Ralstonia mannitolilytica* is commonly found in plants, soil, and water sources. Since *Ralstonia* species are resistant to many antimicrobials, such as carbapenems, treating an infection with *R. mannitolilytica* is challenging. We present an unusual case of a five-month-old infant with a posterior urethral valve whose urine culture showed the growth of a rare organism called *R. mannitolilytica*. UTIs associated with *R. mannitolilytica* are still relatively uncommon. With its high mortality rates, despite antibiotic treatment, this unusual organism needs to be considered in the immunocompromised population presenting with rapidly progressing infections.

## Introduction

Urinary tract infections (UTIs) comprise a common infection seen in children of all age groups [1]. The infection can impact either the lower urinary tract (called cystitis) or the upper urinary tract (called pyelonephritis). As children don’t usually present with the typical symptoms of burning or painful micturition, it is often difficult to differentiate between pyelonephritis and cystitis, particularly in newborns and younger children, solely based on symptoms [2,3]. UTIs are found to be most common during infancy. The signs and symptoms are usually nonspecific during this period, and most infants present with sepsis. In the first year of life, UTIs have an incidence of approximately 0.7% in girls and 2.7% in uncircumcised boys. However, in the case of febrile infants, the incidence changes to 5% and 20%, respectively. After the first year of life, girls are more prone to develop UTIs. UTIs in children follow a bimodal onset, with the first peak at the first year and the second peak between two and four years of life. The recurrence of UTIs is quite common (30% to 50%) and is more common in girls [4,5]. In females, the first infection usually occurs by the age of five years, with peaks during infancy, toilet training, and the onset of sexual activity. *Escherichia coli* is responsible for more than 70% of UTIs, according to the Indian Society of Paediatric Nephrology, followed by *Klebsiella*, *Proteus*, *Enterobacter*, and *Pseudomonas* [1]. *Proteus* infections are found to be more common in boys, and *Staphylococcus saprophyticus* infections are found to be more common in sexually active girls. Anomalies in the urinary tract predispose individuals to UTIs, especially with *Staphylococcus aureus*, *Staphylococcus epidermidis*, and *Haemophilus influenzae*. Rare causes include *Mycobacterium tuberculosis*, *Streptococcus*, fungi, and viruses [3].

*Ralstonia mannitolilytica*, formerly known as *Pseudomonas thomasii*, is an environmental organism found in soil and water. It is a gram-negative, non-fermentative, oxidase-positive bacillus that has recently emerged as an opportunistic pathogen causing infections in the immunocompromised [6]. It can endure in environments with low nutrient levels and even in the presence of disinfectants [7]. It has also been implicated in nosocomial epidemics resulting from contaminated water, peripheral solutions, medical devices, or equipment. Meningitis, pneumonia, and central line-associated infections are among the infections linked to *R. mannitolilytica* in the healthcare setting. Hospital epidemics involving *R. mannitolilytica* have been reported, primarily as bloodstream infections [8]. Although water is the main source, tainted oxygen delivery devices, multi-dose saline bottles, and contaminated disinfectants have also been connected to outbreaks [7,8]. It is also an infection that affects patients post-transplantation during the period of immunosuppression. There is an increasing trend toward reporting *Ralstonia* even in immunocompetent children. Being an indolent organism, a lack of clinical suspicion, and difficulty in growing in routine culture media makes it more difficult to suspect the organism before ruling out others. Hence, often the diagnosis is made after the disease peaks or becomes complicated or serious.

Being a multi-drug-resistant organism makes it easy for it to escape from common antibiotics prescribed, and hence evades the effects of antibiotics. As it complicates the anatomical or physiological complications of the urinary tract, like posterior urethral valve, the effectiveness of antibiotics becomes limited. Hence, often clinicians have to wait until culture and sensitivity reports become available, by which time the patients' condition may deteriorate. The reporting of this case has to be considered in the context of a common hospital contaminant becoming a cause of serious infection, especially in children with anatomical defects of the urinary tract. *Ralstonia* species are resistant to a variety of antimicrobial drugs, including carbapenems, making it a difficult infection to treat [9]. Since *R. mannitolilytica* can produce a biofilm, it remains protected against a large number of antibiotics and disinfectants [9]. We report a rare case of a UTI caused by *R. mannitolilytica* in a five-month-old boy with a background of posterior urethral valve.

## Case presentation

**Figure 1:**
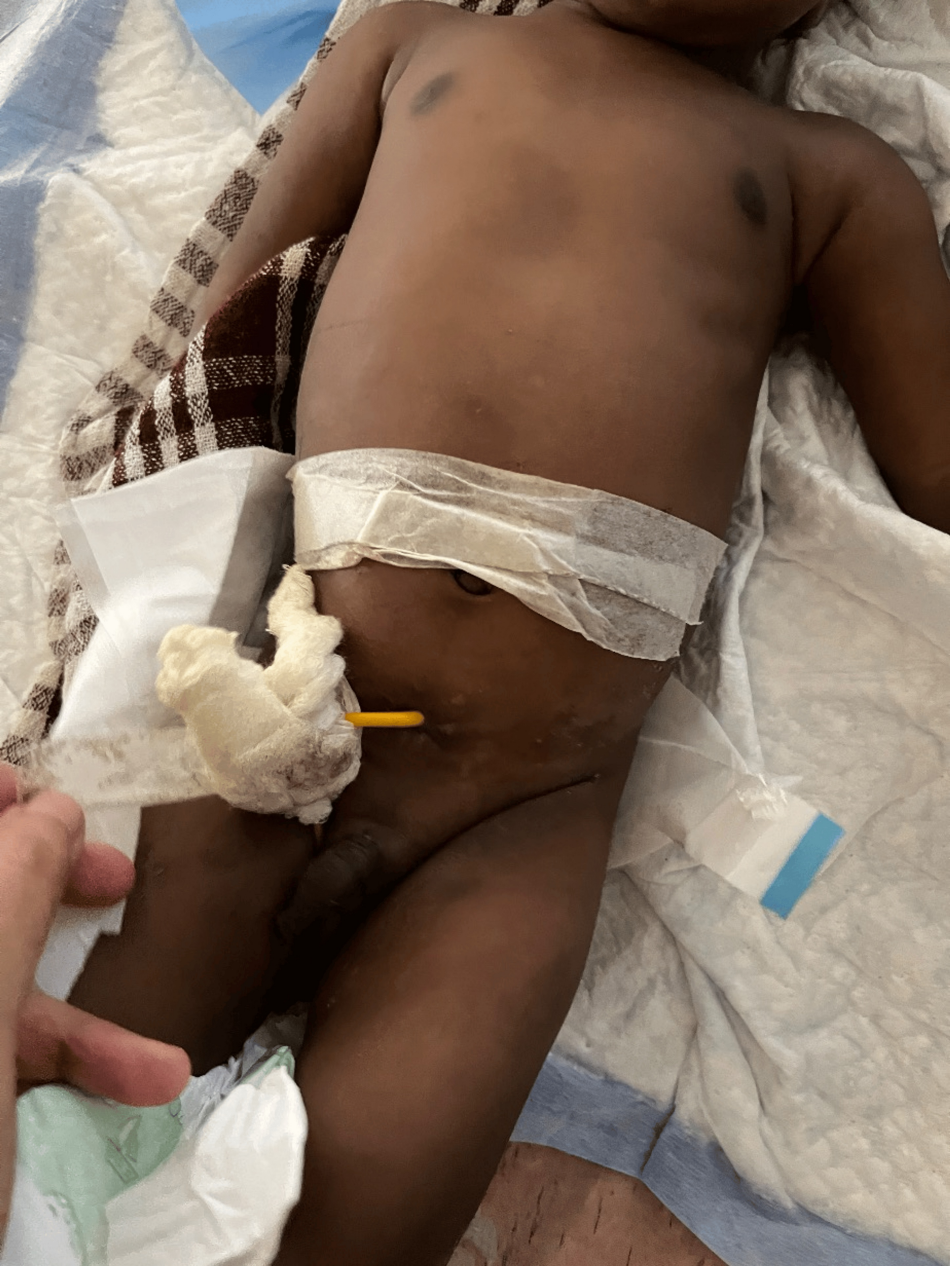
Post-Blocksom's vesicostomy This is a post-operative image of the child. Fulguration was attempted but failed, following which a vesicostomy was done.

A five-month-old male child was brought in with complaints of high-colored urine, noticed by the mother. The child did not cry while micturating and had decreased urine output and vomiting. This child was antenatally diagnosed with gross fetal ascites and bilateral pelvicalyceal system dilatation. The child was born at term with a birth weight of 2600 grams. On day 3 of postnatal life, a screening ultrasound was done, which showed bilateral grade IV hydronephrosis. A micturating cystourethrogram was done, which did not show any vesicoureteral reflux. A cystoscopic examination was also performed, which revealed a posterior urethral valve. Fulguration was attempted, but due to bleeding, the procedure was abandoned, and a vesicostomy was done, as shown in Figure 1.

The post-operative period was uneventful, and the child was treated with antibiotics for 10 days. At discharge, the child was started on uroprophylaxis with cephalexin. At two months of age, the child was readmitted for stoma closure surgery. A urine culture done at the time showed a growth of *Klebsiella pneumoniae*, which was treated with appropriate antibiotics.

**Figure 2:**
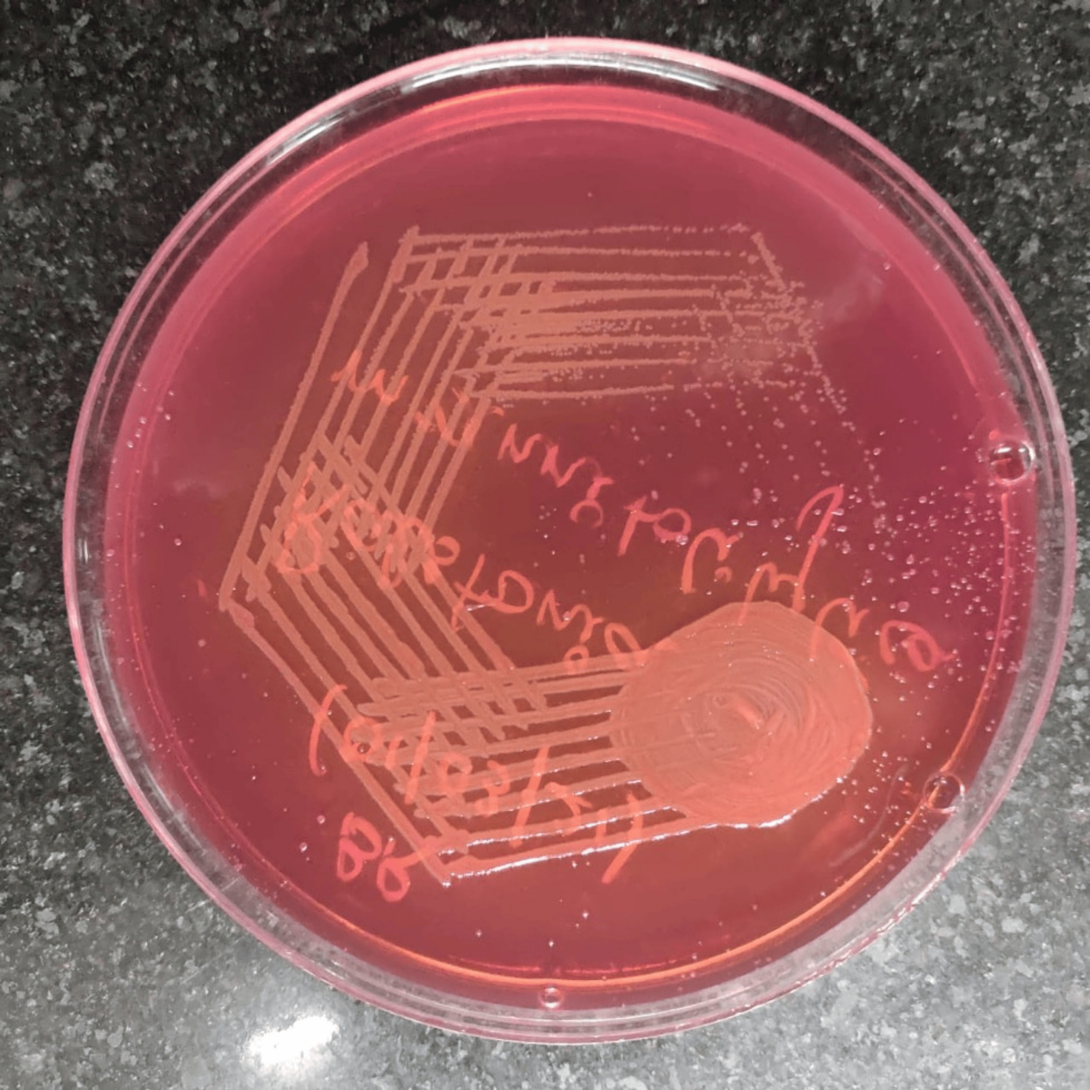
Ralstonia mannitolilytica isolated from urine This MacConkey agar culture plate shows the growth of *Ralstonia mannitolilytica* from the urine sample of the patient as non-pigmented, round to oval colonies.

The child was readmitted at five months with complaints of fever for five days, discoloration of urine, and failure to thrive. Anthropometric indices were all found to be below the third centile for age and sex. Systemic examination was found to be unremarkable at the time of admission. A catheterized urine sample was collected under aseptic precautions and sent for culture. Blood samples were also collected and sent for culture. The blood culture revealed growth of methicillin-resistant *Staphylococcus aureus* (MRSA) sensitive to vancomycin. A day later, the urine culture reports showed the growth of an unusual organism, namely, *R. mannitolilytica*, with a growth of 100,000 CFU/mL, as seen in Figure 2. The culture and sensitivity were tested by the disc diffusion method with MacConkey agar to determine antibiotic sensitivity.

It was sensitive to cefoperazone-sulbactam, piperacillin-tazobactam, and cotrimoxazole. The organism showed resistance to most other antibiotics. Based on the sensitivity pattern, the child was started on IV cefoperazone-sulbactam. Fever spikes settled after 48 hours of starting antibiotics. The antibiotic sensitivity pattern showed maximum resistance to amikacin (64%). Antibiotics were continued for a period of 21 days until the urine culture came back negative.

## Discussion

Since *R. mannitolilytica* was first isolated in 1995, cases have been reported in newborns, cancer patients, dialysis patients, and recipients of renal transplants. *R. mannitolilytica* primarily affects immunocompromised populations, including those with solid organ malignancies, hematological malignancies, post-stem cell transplants, children in intensive care units, or those with central venous catheters. It infects humans through water sources [10]. In hospitals, sources that act as contaminants are water used for dialysis, sterile water for injections, and oxygen delivery systems. *Ralstonia* is also known to survive in saline and chlorhexidine solutions [11]. It is a rare pathogen, with very few cases reported in children. The few reported cases have involved infections with the sister genus, *Ralstonia pickettii* [12]. In the reported cases so far, the primary symptoms were fever and chills. The disease usually progressed rapidly and went on to involve any organ, leading to pneumonia, meningitis, bacteremia, and sometimes septic shock [10]. This index case was unusual, as the child presented with fever and discolored urine and did not show rapid disease progression. The child was relatively stable throughout the hospital stay.

In a study conducted by Kim et al., 18 patients with culture-confirmed *R. mannitolilytica* were identified over a period of 14 years. Among these patients, 77% of cases were identified in the intensive care unit, and most patients presented with pneumonia, with 15 cases showing a positive endotracheal sputum or bronchoalveolar lavage culture. This organism was not identified in urine in any of the cases, unlike in this study, which is what makes our case unusual. Most of the patients had an underlying disease, with hematopoietic stem cell transplant being the most common risk factor, seen in 55% of cases [13]. Lampropoulos et al. reported a case of late-onset sepsis in a premature infant in his fourth week of life. The child presented with sudden clinical deterioration and was started on broad-spectrum antibiotics after sending appropriate cultures. The blood culture identified *R. mannitolilytica* resistant to meropenem and gentamicin [14].

Although there have been few investigations into the processes underlying *R. mannitolilytica*'s antibiotic resistance, it has been noted that the species carries chromosomes containing class D beta-lactamases, including OXA-22 and OXA-60, which are associated with intrinsic resistance [13]. Despite treatment with antibiotics that showed in vitro susceptibility, high mortality rates were noted. According to Daxboeck et al., 12 of the 30 isolates that were isolated became resistant to the antibiotic carbapenem [15]. Every strain in the study conducted by Said et al. was imipenem-sensitive but meropenem-resistant [16]. For *Ralstonia* infection, there is no established empirical treatment, and there is little information available regarding the duration of antibiotic treatment. *R. mannitolilytica* has varying susceptibilities to ceftazidime, cefepime, carbapenem, and aminoglycosides, but it is inherently resistant to colistin. In a study conducted by Kim et al., 95% of cases were found to be resistant to meropenem. An average duration of nine days was noted from the first antibiotic administration to culture negativity, while our case took a longer duration of 21 days to attain culture negativity in the urine sample. *R. mannitolilytica* infection contributed to death in seven patients, of whom five experienced persistent *R. mannitolilytica* infection until death, despite in vitro susceptible antibiotics [13].

This case illustrates a highly uncommon type of UTI caused by *R. mannitolilytica*. It is not common for *R. mannitolilytica* to cause UTIs. This example shows that, although *Ralstonia* infections are uncommon, they should be taken seriously and treated carefully, particularly in young patients.

## Conclusions

This case report highlights a very rare cause of UTI, especially in children. It is known that UTIs do not present with typical symptoms in infants and in the immunocompromised population. Atypical organisms like *Ralstonia* may be missed or ignored in culture by attributing them to contaminants. This fact needs to be borne in mind while dealing with cases that have undergone invasive surgical procedures like ours. Antibiogram-based administration of antibiotics should be strongly considered due to the high mortality rates associated with this infection. The cause of increased mortality with *Ralstonia* infections needs further investigation to find contributing factors other than the immunocompromised state.
